# Does use of a virtual environment change reaching while standing in patients with traumatic brain injury?

**DOI:** 10.1186/1743-0003-10-76

**Published:** 2013-07-16

**Authors:** Amanda Y Schafer, Ksenia I Ustinova

**Affiliations:** 1 Department of Physical Therapy, Central Michigan University, Mount Pleasant, MI, USA

**Keywords:** Virtual reality, Arm-postural coordination, Visual perception, Rehabilitation, Balance, Postural control

## Abstract

**Background:**

Although numerous virtual reality applications have been developed for sensorimotor retraining in neurologically impaired individuals, it is unclear whether the virtual environment (VE) changes motor performance, especially in patients with brain injuries. To address this question, the movement characteristics of forward arm reaches during standing were compared in physical and virtual environments, presented at different viewing angles.

**Methods:**

Fifteen patients with traumatic brain injuries (TBI) and 15 sex- and age-matched healthy individuals performed virtual reaches in a computer-generated courtyard with a flower-topped hedge. The hedge was projected on a flat screen and viewed in 3D format in 1 of 3 angles: 10° above horizon (resembling a real-world viewing angle), 50° above horizon, or 90° above horizon (directly overhead). Participants were instructed to reach with their dominant hand avatar and to touch the farthest flower possible without losing their balance or stepping. Virtual reaches were compared with reaches-to-point to a target in an equivalent physical environment. A set of kinematic parameters was used.

**Results:**

Reaches by patients with TBI were characterized by shorter distances, lower peak velocities, and smaller postural displacements than reaches by control individuals. All participants reached ~9% farther in the VE presented at a 50° angle than they did in the physical environment. Arm displacement in the more natural 10° angle VE was reduced by the same 9-10% compared to physical reaches. Virtual reaches had smaller velocity peaks and took longer than physical reaches.

**Conclusion:**

The results suggest that visual perception in the VE differs from real-world perception and the performance of functional tasks (e.g., reaching while standing) can be changed in TBI patients, depending on the viewing angle. Accordingly, the viewing angle is a critical parameter that should be adjusted carefully to achieve maximal therapeutic effect during practice in the VE.

## Introduction

Virtual reality (VR)-based games and environments are recognized as an effective therapeutic approach in rehabilitation of individuals with acquired brain and spinal cord injuries. A key component of VR applications is their ability to model almost any type of environment and to manipulate visual perception, thereby influencing motor performance. Many studies have reported positive short- and long-term VR practice-related improvements in patients with stroke [1,2], brain and spinal cord injuries [3,4], cerebral palsy [5,6], and Parkinson disease [7]. However, there is no common opinion on how the virtual environment (VE) modifies movement performance, especially in individuals with traumatic brain injuries (TBI).

TBI survivors constitute one of the largest groups of people with disability worldwide [8]. Surprisingly this population has never been a primary target for developing and testing VR applications, with most studies being generally exploratory [9]. Common post-TBI deficits include, but are not limited to, abnormal gait, instability while standing and walking, arm and truncal ataxia, and lack of manual dexterity [10-13]. In addition, more than 50% of patients with severe brain injury have some form of visual perceptual deficiency [14,15], caused by impairment of visual discrimination, visual memory, visual spatial relations, or visual motor integration [16]. These comorbidities complicate the implementation of VR technology in the rehabilitation of patients with TBI. They also raise the question of whether patients with TBI are able to change movement performance in VEs compared to an equivalent physical world.

Using a series of experiments with arm reaching, Levin’s group [17,18] showed that movements in VEs are similar to those made in the equivalent physical world in patients with post-stroke hemiparesis. In contrast, other studies have demonstrated significant differences between the center of pressure displacements during lateral arm-reaching tasks, performed by older adults while standing in the real environment versus a VE delivered on a flat screen [19]. Various personal and technical factors may underlie such contradictory outcomes, including differences in measurement techniques, experimental tasks, and designs. Nevertheless, this contradiction does not help us to understand how to make practice in the VE more beneficial, nor does it elucidate what environmental parameters should be manipulated to improve performance.

We previously showed that the angle at which the VE is presented to participants can be a critical visuomotor variable modulating motor performance and improving outcomes [20]. Participants in the study, when pointing to a flower in a virtual courtyard, reached farther forward when the flower was presented at particular viewing angles. This effect was observed in young healthy individuals. Whether the same mechanism of visuomotor integration remains intact after a brain injury is unclear. Investigation of this question is important in terms of advancing virtual rehabilitation in individuals with TBI.

Considering the importance of rehabilitation for patients with TBI, we designed the current study with two goals. The first goal was to investigate whether the VE changes motor performance in patients with TBI. This question was addressed by comparing the performance of arm reaching in a VE and in an equivalent physical environment (PE) in participants with TBI and healthy individuals, as control. As an experimental task, functional reaching-to-point to a target while standing was chosen. This movement is an essential part of many daily life activities and resembles the functional reach tests commonly used to predict fall risk and to track rehabilitation progress for individuals with neurological deficits [21,22]. The second goal was to test an effect of viewing angle of the VE projection on movement performance in patients with TBI. We hypothesized that depending on the viewing angle, patients with TBI and healthy participants will be able to reach farther in the VE, compared to the reaching in the PE. Altogether, these results can be potentially used for designing VR-based exercises for patients with TBI.

## Methods

### Subjects

Fifteen individuals with TBI (TBI group, 9 females) with a mean (± SD) age of 35.3 ± 11.8 years, and a volunteer sample of 15 healthy sex- and age-matched individuals without known neurological, orthopedic, or cognitive deficits (control group, 8 females) of 33.4 ± 9.1 years participated in the study. Participants in both groups were matched in terms of height, with a mean (± SD) of 167 ± 5.8 cm in the TBI group and 169 ± 5.3 cm in the control group. Eleven participants in the TBI group and 14 participants in the control group were right-handed, according to self-report. All participants signed an informed consent form that was approved by the Institutional Review Board and prepared in compliance with the Declaration of Helsinki.

Participants in the TBI group had different severities of post-TBI impairments. Clinical scores and demographic information are presented in Table 1. Participant scores on the Ataxia Test by Klockgether [23] ranged from 3 to 19 points (mean ± SD: 7.2 ± 4.2 points). The Ataxia Test rates ataxia of gait, stance, upper and lower extremities, dysdiadochokinesis, intention tremor, and dysarthria according to a 6-point scale, with 0 indicating no symptoms and 5 indicating the most severe symptom manifestations in each testing category. A total score of 1–7 corresponds to mild ataxia, 8–21 to moderate ataxia, and 22–35 to severe ataxia. A score > 30 indicates a total inability to perform any of the tested activities.

**Table 1:** Demographic information and clinical and neuropsychological tests scores for participants with TBI

**N**	**Gender**	**Years since TBI**	**Age years**	**Ataxia (_/35)**	**BBS (_/56)**	**FGA (_/30)**	**FM (_/66)**	**MVPT**	**ROCF (_/34)**
S1	M	7	29	19	44	8	61	64	2
S2	M	4	41	3	45	24	65	78	25
S3	M	6	41	12	39	10	66	91	32
S4	F	11	61	6	46	17	66	140	34
S5	F	1.5	34	5	48	21	66	84	27
S6	F	10	29	3	55	29	66	57	27
S7	M	4	22	5	52	27	66	110	31
S8	F	10	38	6	55	21	66	74	28
S9	F	8	44	5	54	27	66	60	16.5
S10	F	10	44	9	48	15	65	78	34
S11	M	10	38	8	49	12	66	55	31
S12	F	.5	18	4	56	27	66	127	33
S13	M	15	38	4	44	27	65	81	33.5
S14	F	2	33	11	43	18	64	68	31
S15	F	1	20	9	53	23	66	97	31
Means ± SD	6 M/9 F	6.6±4.3	35.3±11.8	7.2±4.2	48.7±5.1	20.4±6.1	65.3±1.3	84.2±26.1	27.7±8.75

Participants had scores ranging from 39 to 56 points (mean ± SD: 48.7 ± 5.1 points) on the Berg Balance Scale (BBS) [24]. A score of 45 points on the BBS indicates an increased risk of falling. Participants showed gait performances ranging from 8 to 29 points (mean ± SD: 20.4 ± 6.1 points) on the Functional Gait Assessment Test (FGA) [25]. A score of 22 points on the FGA indicates a high fall risk. Participant scores on the BBS and FGA indicated moderate balance impairments. Overall, 7 participants had mild sensorimotor deficits, and 8 other patients had moderate symptom manifestations. Participants showed nearly full ranges of motion and nearly normal muscle strengths in the major muscle groups. Arm functions were evaluated with the Arm and Hand section of the Fugl–Meyer stroke assessment scale (FM) [26] with the scores ranging from 61 to 66 points, where a score of 66 corresponds to normal functioning.

The visual perceptual abilities of patients were evaluated with a series of neuropsychological tests. Individual scores for each test are shown in Table 1. Visual perception ability was evaluated with the Motor-Free Visual Perception Test (MVPT) [27]. This test measures visual discrimination, visual memory, visual spatial relations, and visual motor integration and is recognized by neuropsychologists as the most sensitive test to detect mild perceptual deficits [19]. A score of 145 on the MVPT indicates the best possible score, and a score of 100 ± 15 points is indicative of average performance. Participant MVPT scores ranged from 55 to 140 points (mean ± SD: 84.29 ± 26.1 points), which represents a low average to mildly impaired performance for the group as a whole. Five participants demonstrated moderate to severe deficits on this measure. 

The Rey-Osterreith Complex Figure (ROCF) test was used to assess visuoconstructive abilities. Participants were instructed to copy a figure from a card onto a piece of paper [28]. The average score of participants in the TBI group was 27.79 ± 8.75 points (range: 2–34 points), with the best possible score being 36. Overall, the average group performance was ≤1^st^ percentile. More specifically, most participants (10/14, 67%) demonstrated visuospatial abilities that were less than or equal to those of the 1^st^ percentile.

### Software and apparatus

The VE was developed with WorldViz software (WorldViz LLC, Santa Barbara, CA, USA). For computer graphics, Alias’ Maya package for 3D animation (Maya, version 7.0.1; Autodesk, Inc., San Rafael, USA) was used. Once developed, the environment was streamed by using a system consisting of a PC (Intel Core 2 Duo Processor, Palo Alto, USA) with a graphics accelerator (nVidia GeForce Go 7300, Santa Clara, USA) integrated with a 6-camera system for motion capture (Qualisys AB, Gothenburg, Sweden). By using real-time captured data, avatars of the hands of each participant were created with 3 markers attached to each hand. The avatars were synchronized with the virtual scenario. The image was projected in 3D format onto an 82-inch screen (1080p Mitsubishi DLP TV bundle, RealD Beverly Hills, CA, USA) and was viewed by the participant in the first-person view via shutter glasses (RealD Professional CrystalEyes 5). An important feature of these shutter glasses is an ability to adjust visual perspective, as the head (and glasses respectively) changes position relatively to the screen. The adjustments in visual perspective are very similar to what people experience in the real world, when observe an object at different viewing angles. The glasses did not appear to interfere with the infrared signal emitted by the motion capture system.

### Experimental procedure

**Figure 1:**
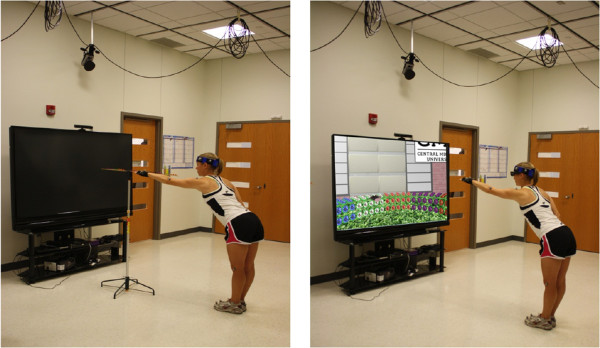
Experimental setup with the control subject reaching forward in the physical environment (A) and in the virtual environment projected onto a screen at 10° (B).

The experimental task consisted of reaching forward while standing in a PE and a VE (Figure 1A-B). The PE task was designed to mimic reaching for flowers within a garden. Instead of flowers, the participant reached for small colored pompons, which were positioned at regular intervals along a meter stick to represent a row of flowers (Figure 1A). The meter stick was suspended horizontally on a stand and was adjusted to the shoulder (specifically, acromion) height of each participant. This device has been validated previously in young and older individuals [29,30]. Participants were instructed to reach forward and point to the farthest pompon possible with their dominant hand, while maintaining balance and without taking a step. This task was repeated 3 times with the dominant arm both before and after the VE reaching task (herein, these tasks are referred to as “PE before” and “PE after,” respectively).

Immediately after PE reaches, participants performed arm reaches in the VE. The VE included a patio surrounded by a semicircular hedge covered with differently colored flowers (Figure 1B). Bands of flowers of different colors helped to distinguish the reaching directions. The presence of the participant was indicated by right and left hands avatars. The projection was designed so that the virtual garden hedge reached approximately shoulder level, with the nearest flower appearing to be at about 90% distance of the arm reaching distance while standing upright. The image was calibrated according to the participant’s height (and arm length respectively) before he/she began the virtual reaching. The visual scene was presented in 1 of 3 equidistant viewing angles: viewing the avatar from “behind” (10° above horizon; Figure 1B), viewing from directly “overhead” (90° above horizon), or viewing from a “middle” view (50° above horizon). An angle of approximately 10° was considered to be the closest to a natural view, similar to what would be used in a real-world garden matching the VR projection. The paradigm is described in details previously [20].

As was mentioned previously, once a participant began bending forward, the angular position of the visual scene was changed slightly to reflect the head displacement as it usually occurs in the real world. The virtual hedge had flowers projected on both its vertical (as if facing the avatar) and horizontal aspects. The size of participant’s hand avatar was scaled, such that coverage of the between-flower distance was equivalent to a distance of 2.5 cm between 2 pompons during real-environment reaches. Reaching was performed with the dominant arm and an open hand, as if the patient were trying to touch the flower rather than pick it. Participants were instructed to reach and point at the farthest flower in a forward direction. Successful pointing was acknowledged by a sound signal, when participant touched the center of the flower. The touch allowed a slight deviation +/− 1 cm in any direction. This deviation included a possible error of +/−0.5 mm, produced by motion capture system. After reaching the flower, if the participant continued to lower the arm, then the hand would appear to “vanish” into the hedge. Participants were instructed to reach for the flower and then return to quiet standing, but not to pass the hand through the hedge. Three reaches were performed in random order in each of the 3 visual angles, for a total of 9 virtual reaches.

All reaches in the PE and VE began with participants standing comfortably with both arms at their sides and feet a comfortable distance apart. Participants were instructed not to change the feet position between trials. Forward reaches involved shoulder flexion (typically beyond 90°) and trunk bending in the sagittal plane.

### Data collection and analysis

During task performance, kinematic data were collected by an optical system for motion analysis (Qualisys AB, Gothenburg, Sweden) at 100 Hz via 28 infrared markers placed on the major bony landmarks of the body. The hand markers were placed on the trapezoid bone, and on the heads of the 2nd and 5th metacarpal bones. Filtered position data (low-pass 8 Hz) from the markers placed on the dominant hand (2nd metacarpals) were used for analysis of the amplitude of the arm endpoint displacement, reach movement time, peak velocity, and time to peak velocity. For each participant, displacement of the center of mass (COM) of the whole body was computed as an averaged mean of all segment COM displacements with the rest of the markers. The segmental COM was calculated with regular anthropometric tables.

The endpoint displacement amplitude was measured as the peak to peak displacement in sagittal plane. The arm movements in the frontal plane and above the shoulder level in the vertical plane were expected to be minimal. The amplitude was expressed in absolute values because the average height of participants was similar between groups.

The reach movement time was calculated from the tangential velocity trace as the time between onset and offset of the endpoint marker displacement. Movement onset and offset were the points at which the velocity rose above or fell but remained below 5% of the maximum peak velocity. The maximum peak velocity was determined in this time window. Time to velocity peak was calculated as percentage of the total reach movement time.

Unless otherwise noted, results are presented as means ± SDs. A mixed two-way ANOVA was used to compare all movement parameters between and within groups. The factors used in ANOVA were group (TBI, control) and experimental condition (PE reaches before and after, and VE reaches at 10°, 50°, and 90° viewing angles). Pearson correlation analysis was used to determine the influence of clinical motor and visual perceptual scale scores on movement parameters in participants with TBI. A significance level of 0.05 was used for all statistical tests.

## Results

### General description

**Figure 2:**
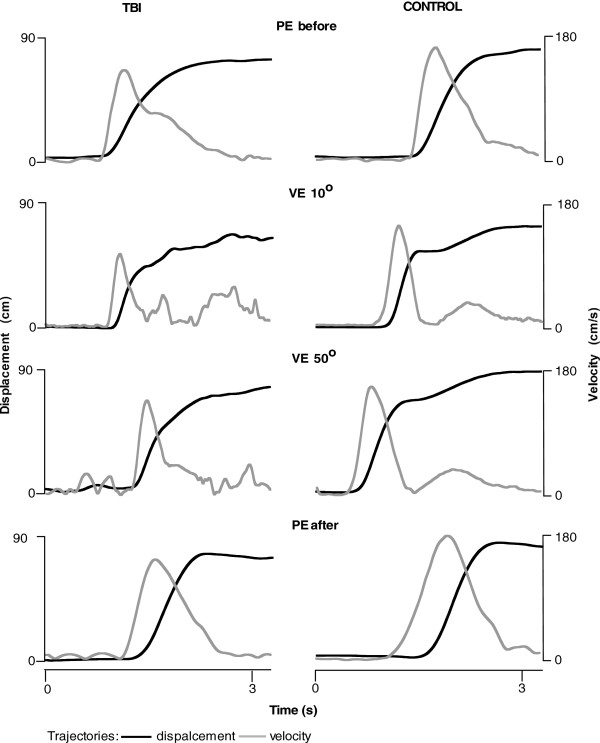
**Trajectories of the arm displacement magnitude (black) and velocity (gray) in a representative participant with TBI (left panel) and in a control individual (right panel).** Reaches were done in the VE presented at 10° and 50° and in the PE before and after virtual reaches.

Figure 2 shows sample endpoint displacement trajectories from one representative participant with TBI (left panel) and one control individual (right panel). Reaches were done in the VE (with the scene presented from the 10° and 50° angles) and in the PE before and after virtual reaches. In all conditions, the control subject reached farther than the subject with TBI. The trajectories in both plots show similar shapes under each condition but differ in movement amplitude. In the VE, participants reached farther when the scene was viewed from the 50° angle. The VE reaches took longer and were characterized by multiple velocity peaks compared to PE reaches, which were performed faster and had a bell-shaped velocity profile with a single velocity peak. In the PE, both participants increased their reaching amplitude at the end, after a series of VE reaches was performed.

**Figure 3:**
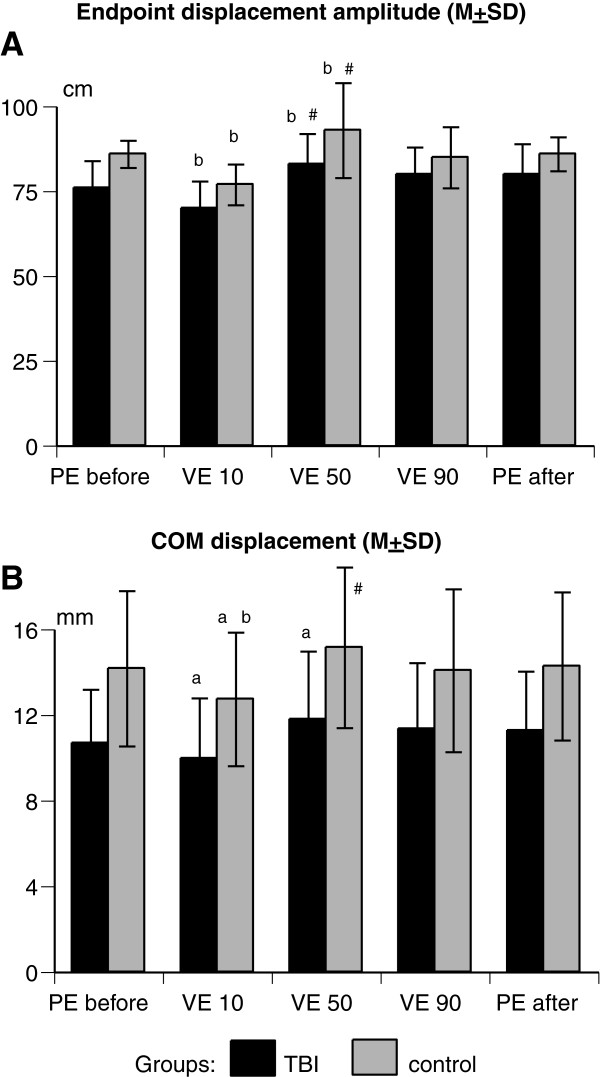
**Means and standard deviations of the endpoint displacement amplitude (A), and center of mass (COM) displacement (B) in participants with TBI (black bars) and in control subjects (gray bars) during reaches in the physical environment before (PE before) and after (PE after) virtual reaches at 10° (VE 10), 50° (VE 50), or 90° (VE 90).** Statistically significant differences were found between reaches with ^a^ PE after vs. VE, ^b^ PE before vs. VE, and ^#^ VE at 10° vs. VE at other angles.

In terms of individual means, control subjects reached farther and showed a greater COM displacement than participants with TBI (Figure 3A-B). A two-way ANOVA revealed significant differences between the groups in terms of the endpoint displacement amplitude (F1,28 = 24.05, *p* < 0.01) and COM displacement (F1,28 = 7.55, *p* < 0.01).

**Figure 4:**
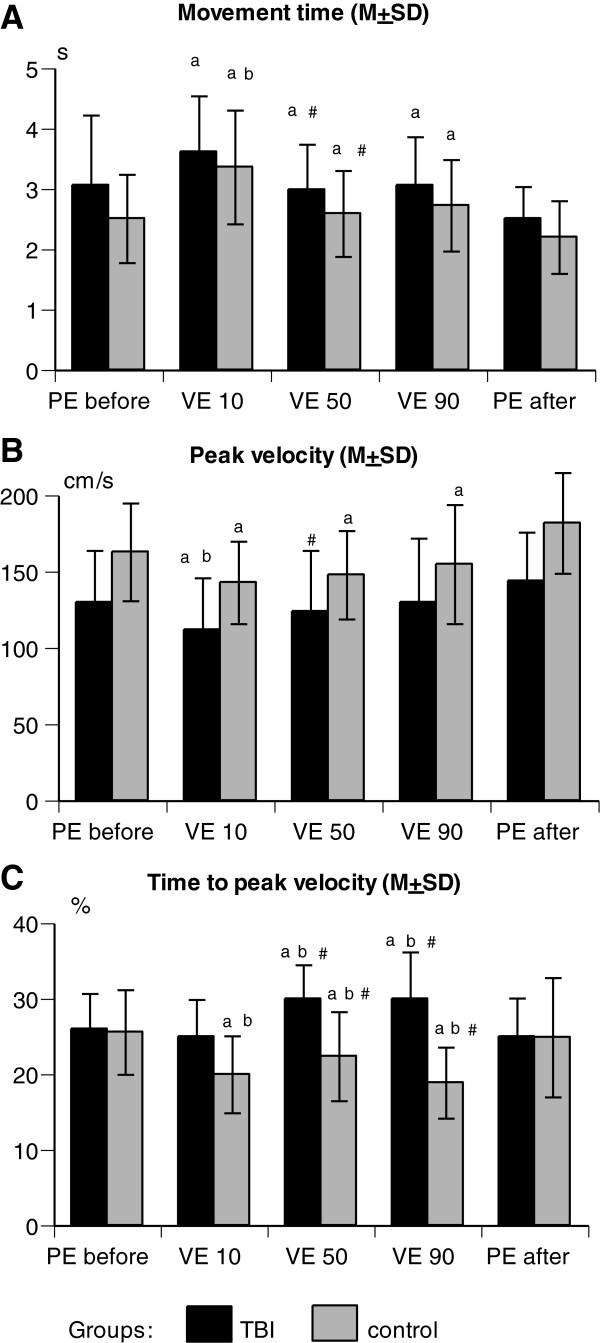
**Means and standard deviations of the movement time (A), peak velocity (B), and time to peak velocity (C) in participants with TBI (black bars) and in control subjects (gray bars) during reaches in the physical environment before (PE before) and after (PE after) virtual reaches at 10° (VE 10), 50° (VE 50), or 90° (VE 90).** Statistically significant differences were found between reaches with ^a^ PE after vs. VE, ^b^ PE before vs. VE, and ^#^ VE at 10° vs. VE at other angles.

Participants with TBI tended to perform reaches more slowly than control individuals (Figure 4A), although the difference in reach movement time was not significant (F1,28 = 2.68, *p* > 0.05). Difference between groups mainly concerned the peak velocity and times to peak velocities (Figure 4B-C). Control subjects performed reaches with greater peak velocity (F1,28 = 8.76, *p* < 0.01) and shorter times to peak velocities (F1,28 = 12.63, *p* < 0.001).

### Virtual versus physical reaches

In both groups, the arm displacement depended on the experimental condition (F14, 112 = 15.45, *p* < 0.001, Figure 3A). The greatest reaching amplitude was achieved in the VE at the 50° angle in both groups (TBI: 83.4 ± 9.1 cm, control: 92.8 ± 14.7 cm). The reaching amplitude in the VE was ~9% farther than arm displacements during PE reaches performed before the VE reaches (TBI: 76.2 ± 8.8 cm, *p* < 0.05; control: 85.9 ± 4.7 cm, *p* < 0.05). When performed at a more natural 10° viewing angle, the VE reaches were about 9-10% shorter than PE reaches in both participant groups (TBI: 70.1 ± 8.1 cm, *p* < 0.01; control: 77.7 ± 6.5 cm, *p* < 0.001).

The COM displacement reflected the endpoint displacement pattern and showed the significant effect of the experimental condition in both groups (F_14,112_ = 12.62, *p* < 0.001, Figure 3B). Participants with TBI showed decreased COM displacement during VE reaches at 10° (9.97 ± 2.81 cm, *p* < 0.05) and increased COM displacement during VE reaches at 50° (11.8 ± 3.8 cm, *p* < 0.05) compared to the COM excursion during PE reaches (10.7 ± 2.5 cm after). Control subjects showed a greater shift of their COM when they performed PE reaches (14.1 ± 3.6 cm before, 14.2 ± 3.46 cm after) compared to VE reaches at 10° (12.7 ± 3.1 cm, *p* < 0.001). Differences between other virtual and physical conditions were insignificant.

In both groups, PE reaches were characterized by decreased movement times (F_14,112_ = 14.58, *p* < 0.001, Figure 4A) and higher peak velocities (F_14,112_ = 8.69, *p* < 0.01, Figure 3B) compared to VE reaches. In the TBI group, differences in peak velocity were found between VE reaches performed at a 10° angle (112.8 ± 34.3 cm/s) and PE reaches performed before (136.6 ± 34.5 cm/s, *p* < 0.01) and after (144.3 ± 32.2 cm/s, *p* < 0.001). Control participants showed lower peak velocities in all 3 VE reaches (10°: 147.0 ± 27.3 cm/s; 50°: 148.4 ± 29.0 cm/s; 90°: 155.3 ± 39.1 cm/s) compared to PE reaches after (182.4 ± 33.4 cm/s; *p* < 0.01). A similar tendency characterized movement time, with all 3 VE reaches taking significantly longer than PE reaches after in both participant groups (both *p* < 0.01). Control participants also showed a difference between the VE reach at 10° and PE reach before (*p* < 0.01) in movement time. Times to peak velocities (acceleration phase) were different tendencies in the TBI and control groups (F_14,112_ = 2.80, *p* < 0.05, Figure 4C). In the TBI group the acceleration phase was prolonged during reaches in VE at 50° and 90° (*p* < 0.05), whereas in the control group time to peak velocity was reduced in all 3 VE conditions (*p* < 0.01) compared to the PE reaches.

Participants in both groups showed farther PE reaches after performing a series of virtual trials. The TBI group increased their reaching distance by ~5% (+4 cm) and reduced their time by ~15% (−0.4 s) during PE reaches after compared to PE reaches before (*p* < 0.05). Participants in the control group showed much more modest improvements compared to the TBI group. They increased arm displacement by ~2% (+1 cm) and significantly increased movement speed by 11-13% (+18.9 cm/s velocity peak and −0.3 s movement time).

### Effect of viewing angle

In VE, both participant groups reached farther when the scene was presented at the middle 50° angle, compared to when the scene was presented at the more natural 10° angle or the overhead 90° position. In the TBI group, compared to reaches at the natural view (10°), VE reaches at 50° were 16% farther (*p* < 0.001), 18% faster, caused 16% greater COM displacement, and 25% prolonged time to peak velocity, (*p* < 0.05). Control participants showed a similar tendency, increasing arm displacement by 17% and COM displacement by 19% during VE reaches at 50° compared to the natural view (*p* < 0.05). The VE reaches at 10° took 23% longer than reaches at 50° (*p* < 0.001). Differences between other VE angle viewing conditions were not significant.

### Clinical characteristics and reaching performance

This study involved TBI participants with a range of Ataxia scores (3–19 points), BBS scores (39–56 points), and FGA scores (8–29 points), as indicated in Table 1. The scores of participants on the neuropsychological tests, measuring visual perceptual abilities, ranged from 55 to 140 points on the MFVT and from 2 to 34 points on the ROCF test. The influence of the motor and visual perceptual deficits on performance of physical and virtual reaches was evaluated with the Pearson correlation coefficient (*r*).

No significant correlation was found between the clinical scores (Ataxia, BBS, FGA) and parameters characterizing performance of VE reaches. Arm displacement and reach movement time during PE reaches moderately correlated with Ataxia score (r = −0.62, *p* < 0.05) and FGA score (r = 0.55, *p* < 0.05). Interestingly, the neuropsychological test scores mainly correlated with VE performance but not with PE reaches. Specifically, the ROCF test scores correlated with displacement magnitude during VE reaches at 50° (r = −0.54, *p* < 0.05) and 90° (r = −0.53, *p* < 0.05), whereas the MFVT scores showed a moderate relationship with the movement time during the same VE reaches (r = 0.62 and r = 0.63, respectively; both *p* < 0.05). No other significant correlations between reaching performance and test scores were revealed.

## Discussion

The TBI group displayed reaches that were characterized by shorter distances, lower peak velocities, and smaller postural displacements than reaches in the control group. All participants reached ~9% farther in the VE presented at a 50° angle than they did in the PE. Arm displacement in the VE at the more natural 10° angle was reduced by the same 9-10% compared to the PE. Virtual reaches were slower than reaches performed in the PE. Overall, the results provide evidence that the VE modifies arm reaching while standing in patients with TBI and in healthy individuals. The environment must be viewed at a particular oblique angle that deviates from the natural view that is typically used to observe the real physical world. In TBI participants, the performance in PE reaches correlated with the severity of motor deficits, whereas visual perceptual abilities affected the performance in the VE.

As a result of the various pathological mechanisms following a brain injury [10-13], a deficit in functional reach characteristics was present in all our participants with TBI. Reaching-to-point movements mostly involve trunk and proximal segments of the body, whereas the manual dexterity requirements are minimal. Thus, the lack of fine hand/finger coordination, which is common among survivors of brain injury, was unlikely to underlie the functional reach deficit. The observed restricted reaching abilities may be attributed to abnormal postural control and to the reduced distance that a person can intentionally displace his or her COM by leaning in a given direction without losing balance, stepping, or grasping a supporting surface. In rehabilitation literature, this ability is referred to as the “limit of stability” and is used as a quantitative measure of postural control [30,31]. The limit of stability is sensitive to even small changes in sensorimotor function [32,33], and affects the performance of daily life activities in vulnerable individuals [34]. The clinical scores on the BBS and FGA, together with reduced COM displacements confirm impairments of postural control in our TBI participants. This can explain their reduction in functional reach abilities compared to healthy individuals. The results are consistent with other studies in patients with stroke [35], multiple sclerosis [36], children with TBI [37], and older individuals [21,30,31].

### Virtual versus physical reaches

Despite the absolute difference in arm displacements, participants in both groups showed increased reaching distance in the VE, although with some reservations. In particular, the virtual scene had to be viewed at an oblique angle for positive outcomes to be observed. In addressing the differences between physical and virtual reaches, this study does not aim to uncover the specific neural mechanism(s) that facilitate the reach-to-point movement in a VE. Because the true mechanisms and potential neural substrates controlling the movements of a body representation (avatar) in an artificially generated 3D environment are not well understood, further explanations of this phenomenon may be considered as speculative. Functional reaches performed in the VE differed from movements made in an equivalent PE. Consistent with the results of previous works [38,39], virtual reaches were slower, had lower peak velocities, and had longer movement times. These features characterize the reaching performance as an exploratory behavior, rather than as an established movement pattern. When exploring a novel environment, an animal as well as human is not yet aware of the task-specific boundaries. In this situation, the final result is unpredictable and may either exceed expectations or be underachieved. Partially supporting this hypothesis, arm reaching in the VE projected in an oblique plane was extremely beneficial for participants, whereas reaching outcomes in other conditions either declined or remained unchanged.

An exploratory nature of movement behavior in a VE may be induced by several factors. Despite recent progress in 3D projection technology, an artificial computer-generated VE distorts visual perception [40]. Observing the environment via goggles typically reduces the field of view, limits the visual resolution acuity, and affects the natural accommodation and vergence mechanisms of the human gaze system, thereby degrading depth cue information [41]. According to previous studies, participants in a VE underestimate distances and perceive objects as being closer than they are [42,43]. Consequently, the user is unsure of the object location in the VE and applies a series of corrective motions, which typically slow down the arm in its approach to a final destination. Confirming this statement experimentally, Subramanian and Levin [44] showed that arm pointing to a virtual target is influenced by viewing media in healthy individuals and patients with stroke. Authors found that viewing the virtual target via head mounted display with reduced field of view changed visual perception accordingly. This resulted in less accurate arm pointing, compared to that performed toward the target projected on a large screen and viewed via polarized glasses. Another factor altering movement performance in VE is a lack proprioceptive feedback. This feedback is necessary for accurate, precise, and predictable reaching-to-point movement [45], which was distorted in our participants as they reached for virtual objects. Finally, the reach-to-point movement in the VE was completed with a hand avatar and not with the index finger as in the PE. Rather than an anatomical extension of the hand of the participant, the avatar is a tool or instrument that needs to be mastered. Motor behaviors associated with complex manual tool use arise from functionally different brain networks, which are typically used for simple reaching and grasping in humans [46]. This explanation may help clarify the VE-induced modification of arm movement in our experiment.

All of the above explanations suggest that our participants might employ different central motor programs during the PE and VE reaches. In the PE, the reaching pattern was formed long before an actual movement started and was based on a subjective estimation by the participant of how far he or she could reach without loss of balance. In other words, the final arm destination was determined before an actual movement began, and few adjustments were applied by the CNS in the process of performance. In the VE, the movement took longer and allowed time for the participant to change his or her final arm position, as well as to modify the performance pattern. The result of adjustment could be either increase or decrease of the reaching distance. These observations suggest that the VE can be a powerful instrument for manipulating human motor behavior, once we learn how to use it efficiently.

Participants with TBI improved their reaches by 7 cm in the VE, and by 4 cm in the PE after performing a series of virtual trials. The reaching increase in our participants fell in the range (3.7–11 cm) of minimal detectable changes established for functional reach tests in vulnerable individuals [47,48]. This fact does not confirm the clinical significance of practicing in a VE, nor does it suggest that practice in VE is more efficient than in the PE. Repetition of the same number of reaches in the PE only could potentially result in an equivalent change. The results of the study do suggest, however that the VE practice can be used as an efficient therapeutic instrument in the rehabilitation of individuals with acquired brain injuries. Successful learning transfer requires that the skills have similar elements, have similar mechanisms of sensory corrections, and be practiced in similar contexts [49]. In this regard, VR-based technologies are the most advantageous in simulating any type of environment, with feedback and sensory conditions closely matching real ones. As another experimental confirmation, after practicing the virtual tasks, participants with TBI showed a significant improvement in their performance of real-world movements, such as pouring a cup of water [50].

Improvements of PE reaches in control individuals were much more modest than those in TBI participants. Most likely, their initial performance was very close to a maximum ceiling that could not be exceeded. Once the ceiling was reached, the control individuals had very little room for improvement, which was not the case in participants with TBI.

### Effect of viewing angle

The second goal of the study was to test the effect of manipulating the viewing angle on reaching distance in patients with TBI. Patients reached farther when the scene was presented at a mid-view angle, whereas reaching distance in the more natural view was shorter. These results support our previous study [20], which utilized the same experimental paradigm and showed that healthy young individuals demonstrate the largest reaches toward a more oblique target (flower). In intact brains, viewing a target under oblique angles changes the activities of the cortical and subcortical areas, which are involved in visuomotor integration [51,52]. Numerous studies have also reported that viewing a target under a more oblique plane alters the estimated object properties and distances [53,54], increases the isometric muscle force [55], and facilitates the performance of cognitive tasks, such as reading a book and watching a display [56,57].

These changes may be mediated by several mechanisms, such as through proprioceptive feedback from extraocular muscles. Finding a target located more obliquely activates muscles that are normally relaxed when looking straight ahead. Through a chain of brainstem reflexes, this proprioceptive feedback modifies the activity of the neck muscles that, in turn, affects postural and motor control [58-60]. Another mechanism altering motor and postural responses may be a different depth perception of the virtual hedge. A hedge presented in a mid-view angle may provide a participant with greater depth perception than the more natural view (10° angle). The consequent changes in eye vergence [61] do not need to be large to influence motion and posture [59]. Finally, the mid-view angles may be more convenient for reaching even in a PE, by providing a better presentation of the properties of the object. This aspect, however, was not investigated in detail.

Overall, the participants with TBI altered their reaching movements in response to the viewing angle in a similar manner as healthy individuals. In the TBI group, the performance of virtual reaches moderately correlated with visual perceptual deficits. These visual perceptual deficits included impaired visual discrimination, visual memory, visual spatial relations, and visual motor integration. The results suggest that the visual perception likely needs to be intact to allow for the maximum efficient use of the VE for motor skills retraining after brain injury. Furthermore, this may be true for a simple goal-directed movement, such as reaching-to-point while standing, but may not include highly coordinated manipulative actions. The lack correlation between motor abnormalities and reaches in the VE, but not in the PE was unexpected. Perhaps the clinical scales were not chosen correctly to reflect the complexity of symptom manifestations in our TBI participants. Another possibility is that virtual and physical reaches require different abilities, and as a result they are affected differently by brain injury-related sensorimotor abnormalities. All these explanations are rather speculative and require further investigation.

### Study limitations

In the present study we mainly tested the effect of VE on arm reaching in patients with TBI, with a potential of using this VE system as a rehabilitation technology. There is an opinion that the virtual rehabilitation system is a valid approach, when causes participants to elicit movements as similar as possible to those produced in the real world. The kinematics of motions was not tested in this study to confirm the statement. Because the end-point of reaching was not determined, it was anticipated that participants may employ different strategies while reaching toward the target. This could affect movement kinematics that is worth of further investigations.

## Conclusion

Our findings confirm the hypothesis that the VE increases reaching distance in patients with TBI, depending on the viewing angle. The results may suggest that visual perception in the VE differs from real-world perception. Accordingly, the viewing angle is a critical parameter that should be adjusted carefully to adapt motor performance and to achieve maximal therapeutic effect during practice in the VE. This observation is important, considering that about 50% of TBI survivors exhibit visual perceptual problems [18,19]. More research needs to be done, with studies including patients with severe sensorimotor and visual perceptual deficits.

## Competing interests

The authors declare that they have no competing interests.

## Authors’ contributions

All authors were equally involved in study design, data collection and analysis, and writing the manuscript. All authors read and approved the final manuscript.
